# Application of machine learning to identify risk factors of birth asphyxia

**DOI:** 10.1186/s12884-023-05486-9

**Published:** 2023-03-08

**Authors:** Fatemeh Darsareh, Amene Ranjbar, Mohammadsadegh Vahidi Farashah, Vahid Mehrnoush, Mitra Shekari, Malihe Shirzadfard Jahromi

**Affiliations:** 1 Mother and Child Welfare Research Center, Hormozgan University of Medical Sciences, Bandar Abbas, Iran; 2 Fertility and Infertility Research Center, Hormozgan University of Medical Sciences, Bandar Abbas, Iran; 3 Endocrinology and Metabolism Research Center, Hormozgan University of Medical Sciences, Bandar Abbas, Iran

**Keywords:** Birth asphyxia, Risk factors, Machine learning

## Abstract

**Background:**

Developing a prediction model that incorporates several risk factors and accurately calculates the overall risk of birth asphyxia is necessary. The present study used a machine learning model to predict birth asphyxia.

**Methods:**

Women who gave birth at a tertiary Hospital in Bandar Abbas, Iran, were retrospectively evaluated from January 2020 to January 2022. Data were extracted from the Iranian Maternal and Neonatal Network, a valid national system, by trained recorders using electronic medical records. Demographic factors, obstetric factors, and prenatal factors were obtained from patient records. Machine learning was used to identify the risk factors of birth asphyxia. Eight machine learning models were used in the study. To evaluate the diagnostic performance of each model, six metrics, including area under the receiver operating characteristic curve, accuracy, precision, sensitivity, specificity, and F1 score were measured in the test set.

**Results:**

Of 8888 deliveries, we identified 380 women with a recorded birth asphyxia, giving a frequency of 4.3%. Random Forest Classification was found to be the best model to predict birth asphyxia with an accuracy of 0.99. The analysis of the importance of the variables showed that maternal chronic hypertension, maternal anemia, diabetes, drug addiction, gestational age, newborn weight, newborn sex, preeclampsia, placenta abruption, parity, intrauterine growth retardation, meconium amniotic fluid, mal-presentation, and delivery method were considered to be the weighted factors.

**Conclusion:**

Birth asphyxia can be predicted using a machine learning model. Random Forest Classification was found to be an accurate algorithm to predict birth asphyxia. More research should be done to analyze appropriate variables and prepare big data to determine the best model.

## Background

Birth asphyxia (BA) is a serious clinical problem worldwide and is a major contributor to neonatal mortality and morbidity [1]. BA is defined as the inability of a newborn to initiate and maintain adequate respiration after birth [2]. According to the world health organization (WHO) Classification of Diseases ICD10, severe BA is present if the APGAR score is 0–3 after 1 min. Mild and moderate birth asphyxia is present when the APGAR score at 1 min is 4–7 [3]. In most developed countries, birth asphyxia accounts for less than 0.1% of newborn deaths. However, it ranged from 4.6/1000 to 7–26/1000 live births in developing countries [1]. BA May cause serious systemic and neurological sequelae due to decreased blood flow and/or oxygen supply to the fetus or infant during the peripartum period [4]. It is also responsible for about a quarter of all neonatal deaths worldwide [5]. BA is one of the top three causes of mortality in children under five (11%), after premature birth (17%), and pneumonia (15%) [6]. According to WHO, 4 million deaths are attributable to BA each year, accounting for 38% of all deaths in children under 5 years of age. In low-income countries, 23% of all neonatal deaths are due to BA [7].

Efforts to improve child health indices have focused on identifying predictors of BA. Both traditional statistical analysis techniques and artificial intelligence (AI) approaches have been used to identify the risk factors of BA. The applications of AI in medicine have increased significantly in recent years. AI in the form of machine learning, natural language processing, expert systems, planning, and logistics methods, and image processing networks offers great analytical capabilities [8]. Machine learning (ML) is a branch of computer science and a branch of AI. These techniques make it possible to derive meaningful connections between data elements from different data sets that would otherwise be difficult to correlate. Due to the large amount and complexity of medical information, ML is considered a promising method to aid diagnosis or predict clinical outcomes [9]. ML can help professionals make decisions, reduce medical errors, improve accuracy in interpreting various diagnoses, and thereby reduce workloads [10]. According to some studies, the use of machine learning methods has been promising in predicting neonatal mortality. For example, Mboya et al. showed that the predictive ability of perinatal death in ML algorithms was significantly superior to the traditional logistic regression method [11]. Therefore, we aimed to use the ML approach to identify the risk factors for BA.

## Methods

This was a cross-sectional study to identify the risk factors of BA. Women who gave birth at Khaleej-e-Fars Hospital in Bandar Abbas, Iran, were retrospectively evaluated from January 2020 to January 2022. Khaleej-e-Fars Hospital is a tertiary hospital with a birth rate of 4000–5000 per year. Data were extracted from the Iranian Maternal and Neonatal Network (IMaN Net), a valid national system, by trained recorders using electronic medical records. Data of all women with singleton pregnancies delivered at the timeline of the study were included in the analysis. Those who gave birth to newborns with congenital anomalies were excluded.

Demographic factors include nationality, age, education level, place of residence, adequate prenatal care (more than six prenatal care visits), smoking status, maternal comorbidities such as anemia, cardiovascular disease, chronic hypertension, pyelonephritis, hepatitis, COVID-19, diabetes, and thyroid dysfunction, drug addiction, and obstetric factors such as gestational, parity, the onset of labor (spontaneous/induced labor/planned cesarean section), preeclampsia, abnormal placentation (placenta previa, placenta accrete), placental abruption, intrauterine growth retardation (IUGR), chorioamnionitis, meconium fluid, fetal presentation, delivery methods, newborn weight, newborn sex, congenital malformation were obtained from patient records.

The primary outcome was whether a machine learning algorithm showed better performance in predicting BA. BA was determined based on a clinical diagnosis from the women’s records using the WHO classification of diseases ICD10 [3].

The following eight machine learning models were used in the study: Logistic regression, Decision Tree Classifier, Random Forrest Classification, XGBoost Classification, Permutation Classification, Feed Forward Deep Learning, Light GBM (LGB), Feed Forward Deep Learning and Support Vector Machines (SVM).

To evaluate the diagnostic performance of each model, six metrics, including area under the receiver operating characteristic curve (AUROC), accuracy, precision, sensitivity, specificity, and F1 score, were measured in the test set. Because AUROC is a widely used index to describe a machine learning model’s ability to predict outcomes [12], we used it as the primary performance metric. The metrics ranged from 0 to 1, with values closer to 1 indicating a better model. The error rate of each model was also analyzed.

The methods for calculating accuracy, precision, recall, and classification error are shown in the equations. Accuracy = (TP)/(TP + FP). In this equation, true positive (TP) represents transactions that were positive and classified as positive. True negative (TN) represents the number of transactions that were negative and classified as positive. False positive (FP) also indicates the number of transactions that were positive and classified as negative. Finally, FN (False Negative) indicates the transactions that were negative and were classified as negative. The equation used to evaluate validity and recall is as follows: Recall = (TP)/(TP + FN) [13]. The F1 value is the harmonic mean of precision and recall. The highest possible value of an F score is 1.0, indicating perfect precision and recall, and the lowest possible value is zero when either precision or recall is zero.

## Results

**Fig. 1:**
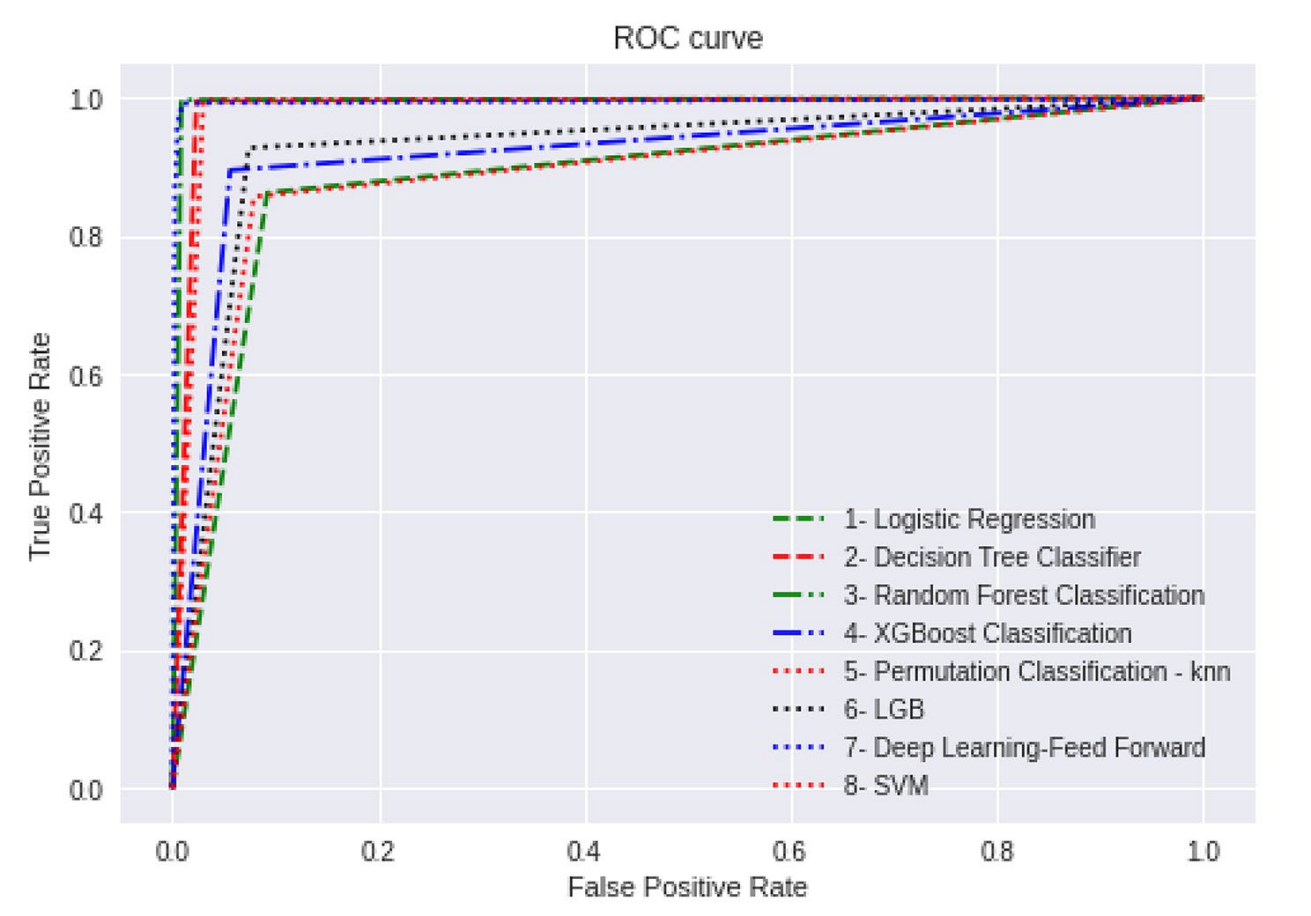
The ROC curves of machine learning models

We found 380 women with a recorded BA out of 8888 deliveries, for a frequency of 4.3%. BA was found in 3.4% of the 5848 vaginally delivered newborns, 10.8% of the 83 vacuum-assisted deliveries, and 5.8% of the 2957 cesarean section newborns. In this study, we attempt to evaluate parameters and feature selection based on performance parameters using various machine learning algorithms. We oversample the dataset using the Adaptive Synthetic (ADASYN) algorithm, then run all of the algorithms in 30- and 70-percentage-point separations of the dataset, plot a ROC chart as shown in Fig. 1 and calculate AUROC as a plot that allows the user to visualize the tradeoff between the classifier’s sensitivity. The accuracy of each algorithm is shown in Table 1. Random Forest Classification, Decision Tree Classification, Permutation Feature Classification with KNN, and Deep Learning were among the most accurate algorithms with an accuracy of 0.98–0.99. Other performance parameters for each algorithm are shown in Table 2. The comparison of performance parameters of different machine algorithms showed that Random Forest Classification is the best model for BA prediction.

**Table 1:** ROC_AUC of machine learning models

Algorithms	ROC_AUC	Accuracy
Logistic Regression	0.88	0.88
Decision Tree Classification	0.98	0.98
Random Forest Classification	0.99	0.99
XGBoost Classification	0.93	0.92
Permutation Feature Classification with KNN	0.98	0.98
Light GBM	0.93	0.93
Deep Learning-Feed Forward	1.0	0.98
SVM	0.88	0.88

**Table 2:** The performance of machine learning models

Algorithms	TP	TN	FP	FN	Accuracy	Precision	Recall	F_1 Score
Decision Tree Classification	1981	1740	51	11	98%	97%	99%	98%
Random Forest Classification	1984	1777	14	8	99%	99%	99.6%	99%
Permutation Feature Classification with KNN	1983	1729	62	9	98%	96%	99%	98%
Deep Learning	1951	1771	41	20	98%	99%	98%	98%

TP: True Positive; TN: True Negative; FP: False Positive; FN: False Negative

**Fig. 2:**
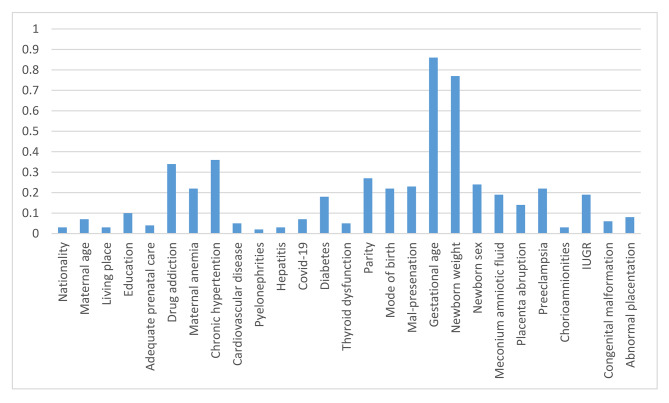
Random Forest Classification Feature Importance

Figure 2 presents an analysis of the importance of variables in the Random Forest Classification algorithm. The importance of the variables revealed that gestational age, newborn weight, newborn sex, preeclampsia, placenta abruption, parity, anemia, and delivery method were considered to be weighted factors.

## Discussion

Despite rapid technological advances, under-five deaths among children remain high. A significant proportion of these deaths worldwide are due to BA. Several studies have been conducted using traditional statistical analysis techniques to identify risk factors for BA. For example, a meta-analysis conducted by Desalew et al. found that maternal illiteracy, prepartum hemorrhage, cesarean section, instrumental delivery, duration of labor, pregnancy-related hypertension, induction of labor, parity, low birth weight, preterm birth, non-cephalic delivery, and meconium staining were significantly associated with BA [14]. Our study was conducted to identify the various factors leading to BA in neonates delivered in a hospital in Bandar Abbas, Iran. According to the findings using the ML approach, Random Forest Classification was found to be the best model to predict BA with an AUC and an accuracy of 0.99. The analysis of the importance of the variables showed that maternal chronic hypertension, maternal anemia, diabetes, drug addiction, gestational age, newborn weight, newborn sex, preeclampsia, placenta abruption, parity, IUGR, meconium amniotic fluid, mal-presentation, and delivery method were considered to be the weighted factors.

Sociodemographic factors were not associated with BA. Among maternal comorbidities, chronic hypertension and diabetes were found to be correlated with BA. Hypertension can lead to a reduction in blood flow and thus asphyxia [15], while diabetes causes intrapartum hypoxia by developing placenta insufficiency [16]. Anemia was also found to be a risk factor for BA, as also observed in previous studies [17, 18]. Maternal anemia is a common pregnancy problem that disrupts maternal and fetal oxygen transport. The disorder may cause fetal hypoxia inside the womb, resulting in BA [19].

Another factor linked to BA was drug addiction. Drug addiction was demonstrated in the current study by declarations from mothers themselves. The actual number of addicts is always several times greater than the number of those identified; however, detecting addicted women is further complicated by their proclivity to conceal and deny the problem. Infants born to addicted mothers are more likely to have prematurity, low birth weight, and IUGR, all of which can contribute to BA [20]. We have found a significant association between gestational age and risk for BA. Preterm birth was found to be one of the most important risk factors for BA, as reported in previous studies [21, 22]. This could be due to the fact that preterm infants face multiple morbidities, including organ system, immaturity, and especially lung immaturity, which leads to respiratory failure [23]. However, some studies have shown that BA increases with gestational age [24, 25]. According to our findings, newborn weight was associated with BA. Low birth weight newborns were at higher risk of developing BA. A potential confounding factor for this could be the fact that mothers of low birth weight babies are often associated with complications such as maternal hypertension and diabetes that occur before conception or before birth [26]. Indeed, many LBW neonates are more likely to be preterm, unable to produce sufficient surfactant, and prone to multiple morbidities, including organ system immaturity, inability to initiate breathing, challenges with cardiopulmonary transition, and eventually developing BA. Fetuses with IUGR who experience growth restriction inside the uterus do not reach their full growth potential for a given gestational age and are at an increased risk of perinatal mortality and morbidity. In IUGR, the reduced rate of fetal growth is essentially an adaptation to an unfavorable intrauterine environment, and it can result in long-term changes in metabolism, growth, and development [27]. Fetuses with IUGR who have intrauterine hypoxia are more vulnerable to asphyxia. BA was observed in 34.4% of IUGR neonates in the clinical setting [28].

Our study showed that parity is associated with BA. The incidence of BA was higher in primiparous mothers. This is consistent with previous studies [29, 30]. Primiparous mothers are more likely to be younger, and they are more likely to have mal-presentations and prolonged obstructed labor. As a result, BA is expected to be more common in these women than in multipara women [31].

Preeclampsia is significantly associated with an increased risk of BA. The finding is in agreement with the evidence [32, 33]. This may be due to the reduction in blood, nutrient, and oxygen supply to the fetus, which may increase the risk of restriction of intrauterine development leading to BA [32].

Placenta abruption was also found to be associated with BA in our study, which was in contrast to previous studies [34]. The association of placenta abruption with BA can be explained by the fact that blood flows from the placenta to the fetus is restricted, leading to hypoxemia and thus asphyxia or stillbirth if maternal transfusion is delayed at the time of delivery [32].

It has long been known that non-cephalic fetuses are at greater risk during the birth process, including asphyxia, birth trauma, and death. This may be because non-cephalic fetuses are more likely to have other problems, such as cord prolapse and head entrapment, that predispose them to BA [35].

Newborns delivered via cesarean section and assisted vaginal birth had a higher rate of BA than those delivered via spontaneous vaginal delivery. This finding is consistent with previous research [36]. This is because either most mothers arrived late due to labor complications or the decision to have a cesarean section was delayed, increasing the burden of BA [37]. Another possibility is that the fetal chest is pressed as the newborns pass through the birth canal, causing secretion to be evacuated. This reduces the likelihood of developing BA, but this physiological benefit is not seen in cesarean section deliveries [38]. Furthermore, both vacuum and forceps extraction exert pressure on the newborn’s brain, which may cause the brain to bleed on the cranium, which contributes to intracranial hemorrhage and BA [39]. This finding suggests that interventions should be carefully evaluated and decided upon during intrapartum care to reduce unnecessary indications for an assisted vaginal birth and cesarean section to reduce the magnitude of BA.

In terms of newborn sex, our findings show that male infants are more likely to develop BA. This is consistent with previous research [40]. Our findings support previous findings of an association between congenital malformation and BA [41]. Although CNS anomalies might be expected to be associated with BA, the presence of other non-CNS birth defects raises important questions about the etiology of BA in these infants.

In line with several previous studies [41, 42], meconium-stained amniotic fluid was associated with BA. Grade III or IV meconium staining has been considered an indicator of a prolonged or severe episode of asphyxia [43]. One possible reason for this could be the inhalation of meconium-stained amniotic fluid, which causes irritation and inflammation of lung tissue or can obstruct the airways, leading to hypoxia and asphyxia at birth. In healthy, well-oxygenated fetuses, this diluted meconium is readily expelled from the lungs by normal physiologic mechanisms, but in a few cases, a meconium aspiration syndrome occurs [44].

The strength of our study is that we used a high-quality registration system in accordance with birth records. We studied both BA after vaginal birth and after cesarean section. We also examined a wide range of clinical factors associated with BA that may not be easily found in registries. Our study was retrospective, which is another limitation. The database did not allow us to determine the exact timing of the different events during pregnancy. For some variables, such as body mass index and weight gain during pregnancy, there was a lack of other data that might influence BA.

## Conclusion

BA can be predicted using a machine learning model. Random Forest Classification was found to be an accurate algorithm to predict BA. Maternal chronic hypertension, maternal anemia, diabetes, drug addiction, gestational age, newborn weight, newborn sex, IUGR, preeclampsia, placenta abruption, parity, meconium amniotic fluid, mal-presentation, and delivery method are risk factors of BA. More research should be done to analyze appropriate variables and prepare big data to determine the best model.

## Data Availability

The datasets generated and/or analyzed during the current study are available from the corresponding author upon reasonable request.

## Abbreviations

AI: Artificial intelligence
BA: Birth asphyxia
IUGR: Intrauterine growth retardation
WHO: World health organization
